# Complications during Pregnancy after Abdominal Burn Scars: A Review

**DOI:** 10.3390/ebj4010005

**Published:** 2023-01-25

**Authors:** Zosha J. van Gelder, Annabel Snoeks, Paul P.M. van Zuijlen, Ralph de Vries, Anouk Pijpe

**Affiliations:** 1 Amsterdam University Medical Center, 1105 AZ Amsterdam, The Netherlands; 2 Burn Center, Red Cross Hospital, 1942 LE Beverwijk, The Netherlands; 3 Department of Plastic and Reconstructive Surgery, Red Cross Hospital, 1942 LE Beverwijk, The Netherlands; 4 Department of Plastic, Reconstructive and Hand Surgery, Amsterdam Movement Sciences, Amsterdam University Medical Center, 1081 HZ Amsterdam, The Netherlands; 5 Pediatric Surgical Centre, Emma Children’s Hospital, Amsterdam University Medical Center, 1105 AZ Amsterdam, The Netherlands; 6 Medical Library, Vrije Universiteit, 1081 HV Amsterdam, The Netherlands; 7 Association of Dutch Burn Centres, Red Cross Hospital, 1941 AJ Beverwijk, The Netherlands

**Keywords:** information, burns, pregnancy, scars, complications, abdominal, foetal

## Abstract

Over the past decades, long-term sequelae of burns have gained increasing attention. Women of childbearing age, who sustained abdominal burns earlier in life, may have unmet information needs on scar-related complications they can expect during pregnancy. We performed a review of the literature to identify abdominal, foetal, and potential other complications during pregnancy in women with abdominal burn scars. PubMed, Embase, and Scopus were searched from inception to 1 July 2020 and updated once on 23 April 2021 (PROSPERO CRD42022187883). Main search terms included pregnancy, scar, burns, and abdominal. Studies on burns obtained during pregnancy have been excluded. Screening, data extraction and bias assessment were conducted by two investigators. We included 22 studies comprising 217 patients. The time between burn injury and first pregnancy varied between 7 and 32 years. Most of the women had normal pregnancies regarding delivery mode and duration of pregnancy. The most reported abdominal burn scar complications were an increased feeling of tightness, itch, pain, and scar breakdown. In some cases, scar release surgery was performed during or prior to pregnancy. Some cases of foetal complications were described. Complications during pregnancy after abdominal burn scars may be limited. More quantitative and qualitative research is needed to assess the maternal and foetal outcomes and complications. The results may be used to inform women and contribute to personalised obstetric management.

## 1. Introduction

**Figure A1:**
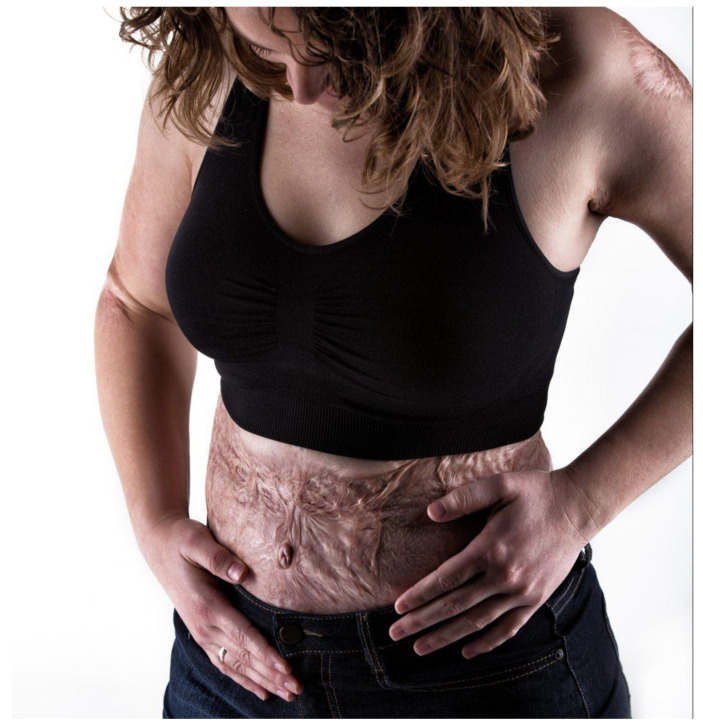
Photo by: Jan van Beijnhem, Foto Studio XL. Written permission from patient and photographer.

Over the years, burn survival has improved in high income countries because there have been numerous improvements in treatments, surgical critical care, a multidisciplinary approach, and surgical interventions [1,2]. The focus of burn care has therefore shifted to survivorship and long-term sequelae of burns, such as scar quality and quality of life [3]. Based on anecdotal input from patients, we learned that women of childbearing age, who sustained severe abdominal burns earlier in life, have unmet information needs on if, when, and which scar-related complications they can expect during pregnancy (Figure A1). Although these women may be a rare subgroup of patients, the impact and long-term consequences can be significant and may even affect their decision to have children. Severe burns of the abdomen may result in scars that may restrict chest and abdominal wall expansion [4]. These scars can lead to multiple complications, such as disfigurement and breathing difficulties.

Pregnancy is accompanied by profound adaptations. This makes pregnant women susceptible to changes in skin and appendages, both physiologic and pathologic, such as infections, probably due to low cellular immunity [5]. Cutaneous alterations during pregnancy are mainly regulated by hormonal, immunologic, and metabolic factors [6]. In the literature there are cases reporting worsening and recurrence of keloids and hypertrophic scars during pregnancy [7,8,9]. It is hypothesised that the hormonal changes stimulate scar growth; however, there is little evidence in the literature to support this [10].

The aim of this study was to identify and describe potential abdominal, foetal, and other complications during pregnancy in women with abdominal burn scars.

## 2. Materials and Methods

### 2.1. Protocol

Our protocol was developed and registered in PROSPERO (CRD42022187883), the international prospective register of systematic reviews [11]. We started our study in 2020 and upon updating our PROSPERO record in 2022, we learned that the PROSPERO system and website had been revised and renewed registration was necessary. For this reason, the first submission and registration date do not match the actual study procedure. For reporting, we followed the Preferred Reporting Items for Systematic Reviews and Meta-Analyses (PRISMA) statement [12].

### 2.2. Search Strategy

To identify all relevant publications, we conducted systematic searches in the bibliographic databases PubMed, Embase.com, and Scopus from inception to 1 July 2020 at first and updated once, using the same search strategy, to include studies between 1 July 2020 and 23 April 2021, in collaboration with a medical information specialist (RdV). The following terms were used (including synonyms and closely related words) as index terms or free-text words: “Cicatrix”, “Scar”, “Pregnancy”, “Maternal”, “Foetal”, “Abdominal”, “Truncal”, and “Burns”. Duplicate articles were excluded. The references of the identified articles were searched for relevant publications. The full search strategies for all databases can be found in Appendix A, Table A1, Table A2 and Table A3.

### 2.3. Selection Process and Criteria

The screening process was conducted with the use of the web-based software platform Rayyan (www.rayyan.qcri.org, accessed on 1 July 2020), which has been selected as a preferred tool by the Cochrane Collaboration. Two reviewers (Z.J.v.G. and A.S.) independently screened all potentially relevant titles and abstracts, and full text articles for eligibility. Differences in judgement were resolved through a consensus procedure involving a third independent reviewer (A.P). We primarily wanted to identify and describe potential abdominal scar complications that may arise during pregnancy in women with abdominal burn scars.

In addition, we wanted to learn what possible foetal and postnatal complications can be expected after abdominal or thoracic burn scars. Studies were included if they met the following criteria: (i) pregnant women; (ii) (abdominal) burn scars due to burn injuries in the past; (iii) foetal complications; (iv) other complications due to burns. We excluded studies if they concerned: (i) burns located solely on extremities; (ii) burns obtained during pregnancy; (iii) languages other than English; (iv) animal studies; (v) editorials and letters. Regarding burns obtained during pregnancy, a recent study was review published which included the presentation of a comprehensive guideline [13].

### 2.4. Data Extraction

Two reviewers (Z.J.v.G. and A.S.) independently performed the data extraction of the included studies. Data were extracted using a standardised data extraction Excel sheet. The extraction sheet contained study characteristics (study design, country, study time period, number of patients, outcome measures, data source), patient characteristics (age during burn, cause of burn, age at pregnancy, percentage of abdomen affected, burn characteristics, duration of pregnancy), and outcomes (abdominal complications, foetal complications, mode of delivery, and breastfeeding). Foetal and postnatal complications were described when multiple studies listed these complications as possible complications in pregnancy.

### 2.5. Risk of Bias Assessment

Risk of bias was assessed using design-concordant tools: the Chambers criteria [14] for case series and case reports and Newcastle–Ottawa Scale (NOS) [15] for the cohort study. Two reviewers (Z.J.v.G. and A.S.) independently evaluated the methodological quality of the full text papers. Differences in judgement of data extraction and risk of bias assessment were resolved through a consensus procedure involving a third independent reviewer (A.P.).

### 2.6. Strategy for Data Synthesis

Since our review was descriptive in nature and we expected a limited number of studies with a wide range of outcomes, we present the extracted data on abdominal burn scar and other complications in a descriptive manner, taking into account the heterogeneity in study design and outcome parameter assessment. For our primary outcome parameter, abdominal burn scar complications, we aimed to use the most widely accepted definition to cover all complications, such as subjective complaints and surgical interventions.

## 3. Results

### 3.1. Literature Search

**Figure A2:**
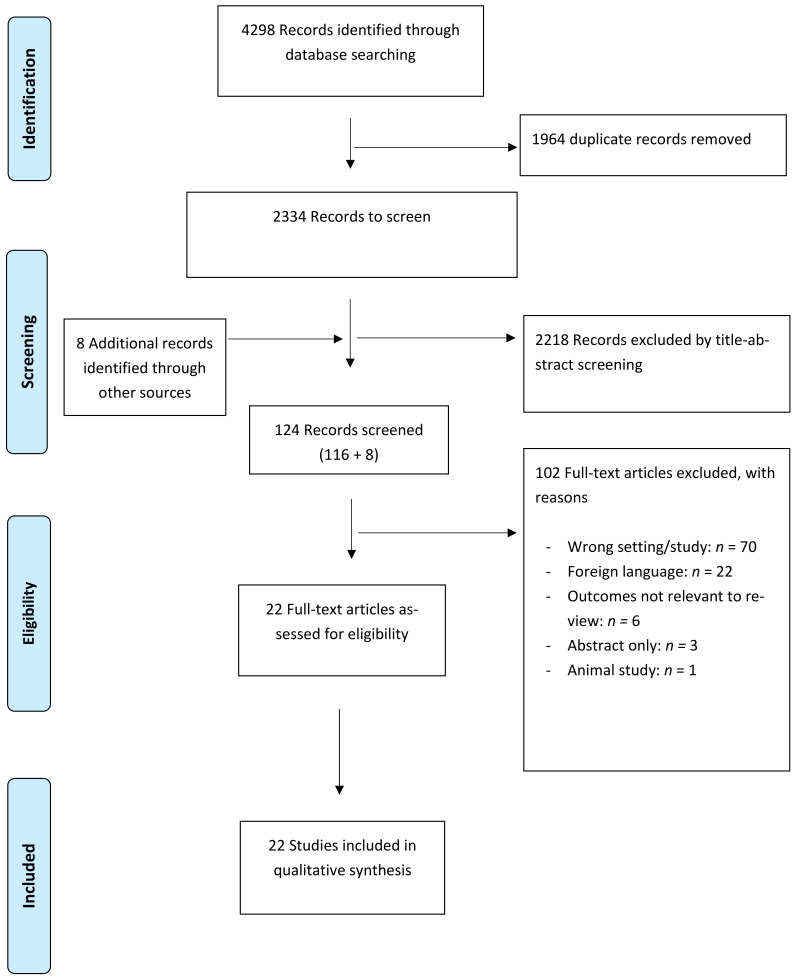
PRISMA flow diagram.

The flow chart of the search and selection process of studies is presented in Figure A2. The literature search generated a total of 4298 references. After removal of 1964 duplicates, 2334 references remained. A search in Cochrane (clinicaltrials.gov and International Clinical Trials Registry Platform, accessed 23 April 2021) showed no additional relevant articles.

A total of 124 full-text articles were reviewed for eligibility including eight articles that were included through a cross reference check. After full-text screening, 22 articles were included. The reasons for excluding were: wrong setting/study (*n* = 70), foreign language *(n* = 22), no relevant outcomes (*n* = 6), no access to the full text (*n* = 3), and animal study (*n* = 1).

### 3.2. Study and Patient Characteristics

Table 1 shows an overview of the study characteristics of the 22 included articles which covered 217 patients, of whom the majority *(n* = 134/217) originated from one observational, comparative registry-based cohort study from Australia. Other studies were retrospective case series (*n*= 7) and case reports (*n* = 14). The first study was published in 1948. Patients originated from all continents.

The risk of bias assessment resulted in 21 out of 22 studies being classified as poor based on the Chambers criteria for case series; the cohort study received seven stars on three domains for nonrandomised studies, which means that the study was of good quality.

### 3.3. Patient and Burn Characteristics

Table 2 describes the characteristics of the patients per study. The 217 patients had a total of 330 pregnancies. The age at burn injury ranged from 3 to 27 years old. Some studies reported only ‘childhood’ for age at burn. The time window between burn injury and, most often, first pregnancy varied between 7 and 32 years. Most patients had a full-term pregnancy. In the studies that reported the Total Body Surface Area burned (TBSA), the range varied from less than 10 percent to more than 90 percent.

Some of the studies wrote about the burns being specifically full thickness burns, second or third degree burns; however, most of the studies did not record the depth of the burns. The burn wound characteristics were mostly unclear: “around abdomen”; “most of the perineum”; “from chest to mid-thigh”.

### 3.4. Abdominal Burn Scar Complications

Abdominal burn scar complications were reported in 14/22 studies (Table 3). The registry-based cohort study did not identify any admissions during pregnancies that were related to any (abdominal, breast, chest wall, back) scar complications or revisions of burn scars or contractures.

The most reported abdominal burn scar complications reported in the other 13 studies were a feeling of tightness, pain, and itch. None of these studies reported on how these complications were assessed, or which measurement instrument or scale was used.

Five studies covering 49 patients had assessed tightness of the scar [6,16,17,18,19]. Approximately half of these patients (*n* = 26/49) indicated having complaints due to this tightness. Treatment for the tightness varied. In almost 84% (*n* = 22/26) of the cases, intervention was not deemed necessary (*n* = 5/26) or was not reported (17/26). Four of the 26 patients were unable to cope at home and were admitted for long-term bed rest.

Five studies covering eleven patients reported women with pain during pregnancy (*n* = 10), without describing details regarding where the pain was located [17,19,20,21,22]. Eight *(n* = 8/11) patients required surgical release due to abdominal pain. The scar release provided immediate pain relief. In one case [19], surgical release caused a very large gap, with a dramatic decompression effect as a result, and premature labour started at 24 weeks of gestation. One day after delivery, the newborn died due to prematurity. In two other studies, one case series and one case report, it was reported that two patients (*n* = 2/13) had pre-existing scar pain before pregnancy [23,24].

Itch of the scars during pregnancy was another complaint. In two studies, 9/22 (41%) patients reported experiencing (intermittent) itch of the scars [16,25]. One patient suffered from a restriction of daily activities because of the itch; therefore, she received ablative fractional laser resurfacing twice during pregnancy, which gave her immediate relief of abdominal tension and itch, increased mobility, and improved respiration.

Other abdominal burn scar complications included minor scar breakdown, reported in 3/27 patients from three studies [6,21,26], for which no intervention was needed. One case report [27] described a patient with mild dyspnoea due to the fact that the lower abdomen distension did not occur and the enlarged uterus was displaced to the upper abdomen; she had to reduce housework.

#### Other Symptoms

A number of single case studies reported symptoms such as a scar-related hot burning sensation [28], backache, nausea, constipation, and dyspepsia [29], where no intervention was reported and the association with burn scars was unlikely.

### 3.5. Foetal Complications

Five case studies described foetal complications, including pressure deformity and foetal death, in women with abdominal burn scars (Table 4). Foetal complications occurred during pregnancy [30,31,32] or after delivery [19,29]. In three of these case reports [30,31,32], it was described that the foetuses showed distress, although in one case (Rajagopalan et al.) it was unclear whether the foetal distress was caused by the abdominal burn scars of the mother. Two of the five foetuses died (Pant, Haeseker); in the case of Pant et al., the mother was already in labour for 22 h upon arrival in the hospital and could not deliver vaginally due to scar tissue, and the foetus had deceased; in the case described by Haeseker and Green, the newborn died due to premature labour after surgical release [19]. Ozog et al. reported on a case in which the foetus had a pressure deformity of the skull which gave the right side of the face a flat appearance and bilateral clubfoot deformity, which was due to little room for rotation and movement during pregnancy [29].

In some of the other studies, foetal complications such as abortions, preterm labour, and a cleft palate were reported; however, the authors did not link this to the patients’ abdominal burn scars.

### 3.6. Other Complications

#### 3.6.1. Delivery Mode

A total of 15 studies reported (*n* = 208) the mode of delivery (Table 5). The registry-based cohort study did not observe a statistically significant difference in mode of delivery between subjects who had sustained a burn to the trunk (partial thickness, full thickness, or unspecified burn depth) and those who had sustained burns to other sites of the body or erythema burns to the trunk [33]. Together with Rai and MacG. Jackson, they conclude that the scarred abdominal wall does not seem responsible for different delivery modes [16,33]. There were several cases described in which the delivery mode was affected by burn scars. Five studies covering twenty-three patients [6,22,28,30,31] reported failure of labour due to cephalopelvic disproportion (*n* = 3/23) or perineal scar tissue (*n* = 4/23).

In two case reports (*n* = 2), foetal lie was transverse at term [21,27]. In the case of Fioretti, the uterus only could expand transversely. Mitsukawa [23] noted that for the delivery method, sufficient extensibility is required in the infra-umbilical skin. In addition, an open leg position is necessary. If the scars cover 50% of more of this skin, caesarean section was desirable. Foetal distress and abdominal pain were two other reported reasons for a caesarean section or induction of labour. In four studies, it was not reported why a caesarean section was required.

#### 3.6.2. Effects on Breastfeeding

Three studies included, covering eight patients, reported that five patients were not able to breastfeed due to damaged breast tissue [17,31,34]. In some cases, the patient was able to lactate from one breast. Two patients had enough lactation to feed their child.

### 3.7. Positive Effects

#### 3.7.1. Scar Improvement

Hormonal changes during pregnancy have been thought to influence scars. Daw (1983) described that none of the six patients included in their case series reported any improvement in their scars [17]. Rai and MacG. Jackson found that 3/22 patients, either seen and examined or reviewed by questionnaire of unknown origin, experienced that the tightness and itching of the scars improved and that the scar became supple after their pregnancies. The rest noticed no change, and none of them said that the scars became worse [16].

#### 3.7.2. Pregnancy as a Natural Tissue Expander

Four case reports described the possibility of using pregnancy after abdominal burns as a natural tissue expander method [21,24,35,36]. In three cases, reconstructive surgery was performed right after delivery to reconstruct an abdomen restricted by old burn scar tissue. In the fourth case, successful scar revision using artificial dermis and split-thickness skin grafting was performed in two stages, nine months before pregnancy. During pregnancy, the grafted skin was extensively and naturally expanded by the gradually growing uterus [24].

## 4. Discussion

This review is the first to investigate potential complications during pregnancy after abdominal burn scars. In this review, we included 22 studies on pregnant women with abdominal scars due to burn injuries in the past. Pregnancies in these women may not be different from pregnancies in women without abdominal burn scars in regard to delivery mode and admissions for scar complications or revisions of burn scars or contractures. However, a certain proportion of these women may experience complaints such as tightness, pain, or itch. In these cases, few severe complications, including the need for surgery, were described. Positive effects have been described as well.

The observation that pregnancies in women with abdominal burn scars do not result in different outcomes compared with those in women who had sustained burns to other sites of the body or erythema burns to the trunk is based on one registry-based cohort study from Australia [33]. This study was based on clinically reported obstetric outcomes covered in the Midwives’ Notification System rather than patient reported complications, such as pain, itch, and tightness. None of the other studies had a comparative nature. An important and interesting finding was that in some women the pregnancy had positive effects, including the experience of more supple scars. Oestrogen makes collagen looser [37]. Because a scar largely consists of collagen, increased levels of oestrogen during pregnancy may have positive effects on the scar, potentially making the scar more supple. In contrast, as mentioned before, in earlier literature, some cases reported worsening and recurrence of keloids and hypertrophic scars during pregnancy [8,9]. There is little evidence in the literature to support this phenomenon. A clinically relevant finding was the use of the expanding belly as a tissue expander. Pregnancy has been used as a tissue expander in the repair of a massive ventral hernia [38], where they used the gravid uterus as an intra-abdominal tissue expander. Although we did not perform a review on tissue expander techniques in women and our results are possibly not complete on this matter, we like to highlight the possibility of using pregnancy as a natural tissue expander method in women with abdominal burn scars; however, of course, ethical considerations regarding the patients’ age should be kept in mind. Sustaining extensive burn injuries at a pre-pubertal age may stunt growth and influence breast formation, and often multiple scar releases are required.

A strength of our study was that it was prioritised questions and concerns raised by women with abdominal burn scars seen in our clinic and those who contacted the National burns information line from the Dutch Burns Foundation, which was one of the reasons we conducted this review. Another strength is the systematic method, which conforms to established guidelines on the conduct and reporting of reviews. Moreover, our literature search was performed in all major databases and was updated (until 23 April 2021) during the study to ensure the inclusion of the most recent studies. The current study also has some limitations. We only included studies written in English. As a result, we may have failed to capture specific scar aspects that are deemed important in non-English speaking countries. We did not use specific search terms (such as postnatal; breasts) which makes it possible that we did not include all possible complications in pregnant women after burn scars; however, this was not the primary aim of our study. The differences in design and outcome measures, such as the site of the burn injury and the severity of the burn, and the poor quality of the studies rendered the possibility of a quantitative analysis of the results, such as the incidence of various types of complications and dependency on burn severity, difficult. Finally, based on the design of the studies, publication bias is likely. The limited amount of evidence, the low quality of the studies, and the heterogeneity in outcomes mean that the findings of this review should be interpreted with some caution.

## 5. Conclusions

In conclusion, in this review, we observed indications that women with abdominal burn scars have normal obstetric outcomes; however, a certain proportion may experience mild to severe complications. Although limited, these data may be used as a first step to better inform women with abdominal burn scars, and it may contribute to personalised obstetric management. Although this perhaps concerns a rare subgroup of patients, the impact of burns on quality of life can be tremendous, even after so many years. The results of this study might also create awareness of the futuristic child wish in the acute treatment of severe abdominal, thoracic, and genital burns in girls and young women. More quantitative and qualitative research is required to assess the accurate incidence, type, and predictive factors of complications during and related to pregnancy in women with abdominal burn scars. This should also include an assessment of foetal outcomes.

## Figures and Tables

**Table 1:** Characteristics of included studies (*n* = 22) by size.

Study	Study Design	Country	Study Time Period	Patients (*n*)	Pregnancies *n*	Outcome Measures ¥	Data Source	Risk of Bias
Duke (2012)	Cohort (retrospective)	Australia	1983–2008	134	213	Abdominal, other	MNS * database	Good
Rai (1975)	Case series (retrospective)	UK	1948–1967	21	42	Abdominal, other	Burns Unit	Poor
Kitzmiller (1998)	Case series (retrospective)	USA	1975–1989	19	31	Abdominal, other	Burns Unit	Poor
Mitsukawa (2015)	Case series (prospective)	Japan	2000–2015	12	-	Abdominal, other	Department of PS ^	Poor
McCauley (1990)	Case series (retrospective)	USA	1967–1985	7	14	Abdominal, other	Burns Institute	Poor
Daw (1983)	Case series (retrospective)	UK	1976–1981	6	11	Abdominal, other	Department of Gynaecology	Poor
Matthews (1982)	Review based on personal communication	UK	Unknown	2	2	Abdominal	Centre for Burns and PS	Poor
Widgerow (1991)	Case report	South Africa	1990	2	2	Abdominal	Department of PS	Poor
Arabi (2019)	Case report	Malaysia	2015	1	1	Other	Health Clinic	Poor
Aykan (2012)	Case report	Turkey	2012	1	1	Abdominal, other	Department of PS	Poor
Cox (2015)	Case report	USA	2015	1	1	Abdominal, other	Department of dermatology	Poor
Del Frari (2004)	Case report	Austria	Unknown	1	1	Natural tissue expansion	Department of PS	Poor
Digregorio (1993)	Case report	USA	Unknown	1	1	Natural expansion	Department of PS	Poor
Fioretti (1987)	Case report	Italy	Unknown	1	1	Abdominal, other	Department of Gynaecology	Poor
Haeseker (1981)	Case report	Wales	Unknown	1	1	Abdominal, foetal	Centre of PS	Poor
Kakagia (2012)	Case report	Greece	Unknown	1	1	Abdominal	Department of PS	Poor
Ozog (1963)	Case report	USA	1962	1	2	Abdominal, foetal	Hospital	Poor
Pant (1995)	Case report	Nepal	Unknown	1	1	Foetal, other	Hospital	Poor
Rajagopalan (2015)	Case report	USA	Unknown	1	1	Foetal, other	Department of Anaesthesiology	Poor
Takeda (2013)	Case report	Japan	Unknown	1	1	Abdominal, other	Department of PS	Poor
Vathulya (2014)	Case report	India	Unknown	1	1	Foetal, other	Department of PS	Poor
Webb (1995)	Case report	Mexico	Unknown	1	1	Abdominal, other	Regional Burn Centre	Poor

* = Midwives’ notification system; ^ = Plastic Surgery; ¥ Other includes: delivery mode, breastfeeding, and scar improvement.

**Table 2:** Overview of patient characteristics.

Study	Age at Burn, Yr	Age at First Pregnancy, Yr	Burn Characteristics	Duration of Pregnancy *
Duke (2012)	Mean 5.7	Mean 20.9	131 patients <10% BSA 3 patients = 10–19% BSA	All: full term
Rai (1975)	Childhood	>15 yr or <45 yr	Full thickness: >4% of the abdomen	35: full term; 2: premature labour; 3: abortion; (2: during pregnancy)
Kitzmiller (1998)	Mean 7.6	^	Mean 55% BSA Full thickness: mean 42%	All: full term
Mitsukawa (2015)	^	^	Mean 64% BSA of the abdomen	^
McCauley (1990)	Mean 7.66	Mean 19.83	Mean 63.21% BSA Full thickness: mean 44.2%	All: full term
Daw (1983)	Mean 6.25	Mean 20.5	Around abdomen	3: full term; 6: induction labour around full term; 2: premature labour
Matthews (1982)	^	^	Circumferential lower abdomen	^
Widgerow (1991)	Case 1: 9 Case 2: childhood	Case 1: 19 Case 2: 21	Circumferential abdomen	Both: full term
Arabi (2019)	5	20	Chest, abdomen, upper limb, and part of her trunk	Full term
Aykan (2012)	4	29	Full thickness of genital region, bilateral lumber areas, lower two thirds of the abdominal wall	Full term
Cox (2015)	2	31	2nd and 3rd degree burns on breasts, abdomen, thighs, lower back	Full term
Del Frari (2004)	14	24	Right lower abdomen, groin area, and thigh	^ (after 8 months)
Digregorio (1993)	27	34	50% BSA third degree burns on face, hands, chest, and abdomen	Full term
Fioretti (1987)	4	24	From lower abdomen to thigh	Full term
Haeseker (1981)	4	21	Full thickness: 60%	Premature labour 3 days after operation
Kakagia (2012)	3	30	Postburn scars torso (anterior + lateral abdominal + chest wall, gluteal areas, breasts)	^
Ozog (1963)	Childhood	19	From chest to midthigh	1st pregnancy: 5 months; 2nd pregnancy: premature
Pant (1995)	5	17	Most of the perineum	^
Rajagopalan (2015)	6	38	>90% BSA on chest, neck, face, abdomen, elbows, knees	27 weeks
Takeda (2013)	6	23	BSA: 80% Full thickness: 65%	36 weeks
Vathulya (2014)	6	22	Chest region to abdomen + perineal region with supra-clitoral hooding deformity; left breast nipple-areolar complex	Third trimester
Webb (1995)	3	23	Full thickness: mean 40%	Full term

* = all pregnancies; ^ = - this information is not provided in the publication.

**Table 3:** Abdominal outcomes.

Study	Complication	Follow Up	Outcome	Notes
Duke (2012)	No admissions during pregnancy for scar complications, revisions of scars or contractures; 2 times hypertrophic/ keloid scar was recorded	-	No long-term detrimental effects of burns on pregnancy, delivery or to the foetus	The majority of trunk burns were burns of partial thickness or unspecified depth
Rai (1975)	2 itch, 7 tightness, 6 both	Unknown	Unknown	Three patients said the scars improved after pregnancy and in subsequent pregnancies
Kitzmiller (1998)	Minor scar breakdown in third trimester 25% instance of subjective sensation of abdominal tightness	Local care No narcotics necessary	Healed rapidly after delivery	
McCauley (1990)	Breakdown of abdominal scar tissue in 3^rd^ trimester	Unknown	Unknown	
Daw (1983)	Tautness with a hot burning sensation to constant indescribable pain	Admitted to hospital, bed rest, inactivity, analgesics, surgical decompression (36 weeks)	Induction of labour in 6 of 11 pregnancies, premature labour	
Matthews (1982)	Maternal pain	Surgical intervention during 3rd trimester	Immediate pain relief	
Widgerow (1991)	Tightness, contracture limited progress of pregnancy	Surgical release (16 weeks/4 months)	Normal expansion of the uterus	
Aykan (2012)	Scar related hot burning sensation in 3rd trimester	Unknown	Unknown	Shortly after the operation, abdominal scar tissue tension-related symptoms and hot burning sensation decreased.
Cox (2015)	Intermittent ich and mild restriction with inactivity	Ablative functional laser (30 and 38 weeks)	Immediate postprocedure relief of tension, increased mobility, and improved respiration	Comfort and functionality were improved compared with prepregnancy; scar contour and pliability had improved
Fioretti (1987)	Mild dyspnoea	Had to reduce housework	Unknown	
Haeseker (1981)	Tightness and pain; potential obstruction for growing uterus	Surgical scar release (24 weeks)	Decompression→ premature labour→ foetus died	
Ozog (1963)	backache, nausea, anorexia, vomiting, dyspepsia, and severe constipation due to direct pressure	Re-examined each month and drug treatment	Refractory to drug treatment	
Takeda (2013)	Abdominal pain	Expansion abdominoplasty (20 weeks)	Abdominal wall expansion and foetal growth were found to be favourable	
Webb (1995	Pain and a localised area of skin breakdown	Close monitoring	38 weeks: pre-eclampsia→ CS	Taking advantage of the natural skin expansion of pregnancy

**Table 4:** Foetal outcomes.

Study	Complication	Cause	Direct/Indirect Cause on Complication	Outcome	Likelihood of Relation to Burns
Haeseker (1981)	Premature labour	Surgical release→ decompression effect	Indirect	Dead due to prematurity	Likely/certain
Ozog (1963)	Pressure deformity of the skull→ right side of the face flat appearance + bilateral clubfoot deformity	Little room for rotation and movement	Direct	Temporary deformities	Likely
Pant (1995)	Non progressive labour (22hours in labour)	Scar tissue on perineum→ oedematous vulva with the foetal scalp visible	Direct	Dead	Likely/certain
Rajagopalan (2015)	Repeated foetal decelerations and non-reactive tracings	Unknown (preeclampsia, elevated aminotransferase, hyperglycaemia?) (placental insufficiency?)	Unknown	Emergency caesarean	Possible
Vathulya (2014)	Absent foetal heart sounds, meconium stained liquor, nonprogressive labour	Unknown	Unknown	Emergency caesarean	Possible

**Table 5:** Mode of Delivery.

Study	Mode of Delivery	Potential Explanation	Notes
Duke (2012)	142: NVD *; 26: instrument; 45: CS ^	Unknown	No statistically significant differences between subjects who had sustained a burn to the trunk and those with burns to other sites of the body or erythema burns to trunk
Rai (1975)	31: NVD; 4: forceps; 2: CS	Scarred abdominal wall was not responsible/Any lack of expulsive force not total excluded: an objective study by measurements of intraabdominal pressure changes and abdominal wall extensibility in relation to cervical dilatation is made	One of the forceps deliveries contained twins. Three abortions
Kitzmiller (1998)	28: NVD; 3: CS	Failure of labour due to cephalopelvic disproportion	Abdominal wall healing after CS was not complicated
Mitsukawa (2015)	2: NVD; 9: CS; 1: Not pregnant yet	If patients have scars covering 75% or more of the total abdominal area, scar release surgery is always performed. In addition, an open leg position is necessary.	
McCauley (1990)	13: NVD; 1: CS	Unknown	1 elective caesarean section
Daw (1983)	All: NVD	Abdominal pain from tightness	In 6 of 11 pregnancies necessitated
Aykan (2012)	CS	perineal scar tissue was dense and preventing vaginal delivery	classical Pfannenstiel incision was preferred
Cox (2015)	CS	Non progressive labour	6 months after delivery she reported negligible tension and itch in the scarred areas
Fioretti (1987)	CS	the uterus could only expand transversely, foetal lie was transverse at term	Elective caesarean section
Ozog (1963)	1st: stillborn, twins; 2nd: forceps	Unknown	1 month premature
Pant (1995)	Incision anterior to the anus up to symphysis in the midline	Scar tissue covered most of the perineum	An incision was made anterior to the anus up to the symphysis pubis in the midline to separate the vulva obstruction.
Rajagopalan (2015)	CS	Foetal distress	Emergency caesarean section
Takeda (2013)	CS	Perineal scar contractures resulted in rigidity of the soft birth canal and limited hip joint flexion	Elective caesarean section
Vathulya (2014)	CS	Foetal distress and supra-clitoral hooding deformity: the clitoris, and the labia anterior 2/3 were almost invisible	Emergency caesarean section
Webb (1995)	CS	Preeclampsia and transverse lie	Caesarean section

* =Normal Vaginal Delivery; ^ = Caesarean section.

## Appendix A

**Table A1:** PubMed Session Results (23 April 2021).

Search	Query	Items Found
#8	#4 OR #7	1388
#7	#5 AND #6	248
#6	“pregnan*“[ti] OR “pregnan*”[ot] OR “vaginal”[ti] OR “vaginal”[ot] OR “abdominal”[ti] OR “abdominal”[ot] OR “truncal”[ti] OR “truncal”[ot]	402,641
#5	“burn”[ti] OR “burns”[ti] OR “scald*”[ti] OR “postburn *”[ti] OR (“thermal”[ti] AND “injur*”[ti]) OR (“chemical”[ti] AND “injur *”[ti]) OR “burn”[ot] OR “burns”[ot] OR “scald*”[ot] OR “postburn *”[ot] OR (“thermal”[ot] AND “injur*”[ot]) OR (“chemical”[ot] AND “injur*”[ot])	44,069
#4	#1 AND #2 AND #3	1153
#3	“abdom*”[tw] OR “truncal”[tiab]	428,002
#2	“Pregnancy Complications”[Mesh] OR “Gestational Age”[Mesh] OR “Pregnancy”[Mesh] OR “Pregnancy Trimesters”[Mesh] OR “Pregnant Women”[Mesh] OR “Preconception Care”[Mesh] OR “Maternal Mortality”[Mesh] OR “Maternal Health”[Mesh] OR “Fetal Mortality”[Mesh] OR “Delivery, Obstetric”[Mesh] OR “maternal”[tiab] OR “mother*”[tiab] OR “fetal”[tiab] OR “foetal”[tiab] OR “fetus”[tiab] OR “foetus”[tiab] OR “maternity”[tiab] OR “pregnan*”[tiab] OR “pseudopregnan*”[tiab] OR “gravidit*”[tiab] OR “nulligravid*”[tiab] OR “primigravid*”[tiab] OR “multigravid*”[tiab] OR “gravidation”[tiab] OR “gravidarum”[tiab] OR “gravida”[tiab] OR “parturition*”[tiab] OR “parity”[tiab] OR “childbirth*”[tiab] OR “birthing”[tiab] OR “birth”[tiab] OR “stillbirth”[tiab] OR “childbed”[tiab] OR (“abdominal”[tiab] AND “deliver*”[tiab]) OR “gestation*”[tiab] OR “parturien*”[tiab] OR “child-bear*”[tiab] OR “childbear*”[tiab] OR “placentat*”[tiab] OR “prepregnan*”[tiab] OR “conception*”[tiab] OR “preconception*”[tiab] OR “obstetric*”[tiab] OR “prenatal”[tiab] OR “perinatal”[tiab] OR “intranatal”[tiab] OR “antenatal”[tiab] OR “prepartum”[tiab] OR “peripartum”[tiab] OR “intrapartum”[tiab] OR “antepartum”[tiab] OR “pre-natal”[tiab] OR “peri-natal”[tiab] OR “intra-natal”[tiab] OR “ante-natal”[tiab] OR “pre-partum”[tiab] OR “peri-partum”[tiab] OR “intra-partum”[tiab] OR “ante-partum”[tiab]	1,625,975
#1	“Cicatrix”[Mesh] OR “cicatr*”[tiab] OR “keloid*”[tiab] OR “scar”[tiab] OR “scars”[tiab] OR “scarring”[tiab] OR “contractur*”[tiab]	123,866

**Table A2:** Embase.com Session Results (23 April 2021).

Search	Query	Items Found
#9	#8 NOT (‘conference abstract’/it OR ‘conference review’/it)	1778
#8	#4 OR #7	2433
#7	#5 AND #6	274
#6	‘pregnan*’:ti OR ‘pregnan*’:kw OR ‘vaginal’:ti OR ‘vaginal’:kw OR ‘abdominal’:ti OR ‘abdominal’:kw OR ‘truncal’:ti OR ‘truncal’:kw	511,336
#5	‘burn’:ti OR ‘burns’:ti OR ‘scald*’:ti OR ‘postburn*’:ti OR (‘thermal’:ti AND ‘injur*’:ti) OR (‘chemical’:ti AND ‘injur*’:ti) OR ‘burn’:kw OR ‘burns’:kw OR ‘scald*’:kw OR ‘postburn*’:kw OR (‘thermal’:kw AND ‘injur*’:kw) OR (‘chemical’:kw AND ‘injur*’:kw)	53,170
#4	#1 AND #2 AND #3	2171
#3	‘abdom*’:ab,ti,kw,de OR ‘truncal’:ab,ti,kw	769,423
#2	‘pregnancy complication’/exp OR ‘gestational age’/exp OR ‘pregnancy’/exp OR ‘named groups by pregnancy’/exp OR ‘prepregnancy care’/exp OR ‘maternal mortality’/exp OR ‘maternal welfare’/exp OR ‘fetus mortality’/exp OR ‘fetal health’/exp OR ‘obstetric delivery’/exp OR ‘maternal’:ab,ti,kw OR ‘mother*’:ab,ti,kw OR ‘fetal’:ab,ti,kw OR ‘foetal’:ab,ti,kw OR ‘fetus’:ab,ti,kw OR ‘foetus’:ab,ti,kw OR ‘maternity’:ab,ti,kw OR ‘pregnan*’:ab,ti,kw OR ‘pseudopregnan*’:ab,ti,kw OR ‘gravidit*’:ab,ti,kw OR ‘nulligravid*’:ab,ti,kw OR ‘primigravid*’:ab,ti,kw OR ‘multigravid*’:ab,ti,kw OR ‘gravidation’:ab,ti,kw OR ‘gravidarum’:ab,ti,kw OR ‘gravida’:ab,ti,kw OR ‘parturition*’:ab,ti,kw OR ‘parity’:ab,ti,kw OR ‘childbirth*’:ab,ti,kw OR ‘birthing’:ab,ti,kw OR ‘birth’:ab,ti,kw OR ‘stillbirth’:ab,ti,kw OR ‘childbed’:ab,ti,kw OR (‘abdominal’:ab,ti,kw AND ‘deliver*’:ab,ti,kw) OR ‘gestation*’:ab,ti,kw OR ‘parturien*’:ab,ti,kw OR ‘child-bear*’:ab,ti,kw OR ‘childbear*’:ab,ti,kw OR ‘placentat*’:ab,ti,kw OR ‘prepregnan*’:ab,ti,kw OR ‘conception*’:ab,ti,kw OR ‘preconception*’:ab,ti,kw OR ‘obstetric*’:ab,ti,kw OR ‘prenatal’:ab,ti,kw OR ‘perinatal’:ab,ti,kw OR ‘intranatal’:ab,ti,kw OR ‘antenatal’:ab,ti,kw OR ‘prepartum’:ab,ti,kw OR ‘peripartum’:ab,ti,kw OR ‘intrapartum’:ab,ti,kw OR ‘antepartum’:ab,ti,kw OR ‘pre-natal’:ab,ti,kw OR ‘peri-natal’:ab,ti,kw OR ‘intra-natal’:ab,ti,kw OR ‘ante-natal’:ab,ti,kw OR ‘pre-partum’:ab,ti,kw OR ‘peri-partum’:ab,ti,kw OR ‘intra-partum’:ab,ti,kw OR ‘ante-partum’:ab,ti,kw	1,958,693
#1	‘scar’/exp OR ‘cicatr*’:ab,ti,kw OR ‘keloid*’:ab,ti,kw OR ‘scar’:ab,ti,kw OR ‘scars’:ab,ti,kw OR ‘scarring’:ab,ti,kw OR ‘contractur*’:ab,ti,kw	167,645

**Table A3:** Scopus Session Results (23 April 2021).

Search	Query	Items Found
#8	#4 OR #7	1132
#7	#5 AND #6	278
#6	TITLE (“pregnan*” OR “vaginal” OR “abdominal” OR “truncal”) OR AUTHKEY (“pregnan*” OR “vaginal” OR “abdominal” OR “truncal”)	498,977
#5	TITLE (“burn” OR “burns” OR “scald*” OR “postburn*” OR (“thermal” AND “injur*”) OR (“chemical” AND “injur*”)) OR AUTHKEY (“burn” OR “burns” OR “scald*” OR “postburn*” OR (“thermal” AND “injur*”) OR (“chemical” AND “injur*”))	64,253
#4	#1 AND #2 AND #3	865
#3	TITLE-ABS (“abdom*” OR “truncal”) OR AUTHKEY (“abdom*” OR “truncal”)	463,391
#2	TITLE-ABS (“maternal” OR “mother*” OR “fetal” OR “foetal” OR “fetus” OR “foetus” OR “maternity” OR “pregnan*” OR “pseudopregnan*” OR “gravidit*” OR “nulligravid*” OR “primigravid*” OR “multigravid*” OR “gravidation” OR “gravidarum” OR “gravida” OR “parturition*” OR “parity” OR “childbirth*” OR “birthing” OR “birth” OR “stillbirth” OR “childbed” OR (“abdominal” AND “deliver*”) OR “gestation*” OR “parturien*” OR “child-bear*” OR “childbear*” OR “placentat*” OR “prepregnan*” OR “conception*” OR “preconception*” OR “obstetric*” OR “prenatal” OR “perinatal” OR “intranatal” OR “antenatal” OR “prepartum” OR “peripartum” OR “intrapartum” OR “antepartum” OR “pre-natal” OR “peri-natal” OR “intra-natal” OR “ante-natal” OR “pre-partum” OR “peri-partum” OR “intra-partum” OR “ante-partum”) OR AUTHKEY (“maternal” OR “mother*” OR “fetal” OR “foetal” OR “fetus” OR “foetus” OR “maternity” OR “pregnan*” OR “pseudopregnan*” OR “gravidit*” OR “nulligravid*” OR “primigravid*” OR “multigravid*” OR “gravidation” OR “gravidarum” OR “gravida” OR “parturition*” OR “parity” OR “childbirth*” OR “birthing” OR “birth” OR “stillbirth” OR “childbed” OR (“abdominal” AND “deliver*”) OR “gestation*” OR “parturien*” OR “child-bear*” OR “childbear*” OR “placentat*” OR “prepregnan*” OR “conception*” OR “preconception*” OR “obstetric*” OR “prenatal” OR “perinatal” OR “intranatal” OR “antenatal” OR “prepartum” OR “peripartum” OR “intrapartum” OR “antepartum” OR “pre-natal” OR “peri-natal” OR “intra-natal” OR “ante-natal” OR “pre-partum” OR “peri-partum” OR “intra-partum” OR “ante-partum”)	1,957,453
#1	TITLE-ABS (“cicatr*” OR “keloid*” OR “scar” OR “scars” OR “scarring” OR “contractur*”) OR AUTHKEY (“cicatr*” OR “keloid*” OR “scar” OR “scars” OR “scarring” OR “contractur*”)	141,659
